# Subtype-Specific mRNA Signatures of Human Ribosomal Proteins in Pediatric Cancers

**DOI:** 10.3390/ijms262412036

**Published:** 2025-12-14

**Authors:** Anshuman Panda, Anupama Yadav, Gyan Bhanot, Shridar Ganesan

**Affiliations:** 1 Perlmutter Cancer Center, New York, NY 10016, USA; ap1022@scarletmail.rutgers.edu; 2 NYU Grossman School of Medicine, New York, NY 10016, USA; 3 Rutgers Cancer Institute of New Jersey, New Brunswick, NJ 08903, USA; 4 Dana-Farber Cancer Institute, Boston, MA 02215, USA; 5 Harvard Medical School, Boston, MA 02115, USA; 6 Departments of Molecular Biology & Physics, Rutgers University, Piscataway, NJ 08854, USA; 7 Rutgers Robert Wood Johnson Medical School, New Brunswick, NJ 08901, USA

**Keywords:** ribosomal proteins, pediatric cancers, medulloblastoma, acute lymphoblastic leukemia, heterogeneous ribosomal composition

## Abstract

A growing body of recent work suggests the possibility of heterogeneous ribosomal composition. We recently observed subtype-specific mRNA and copy number variation signatures of human ribosomal proteins (RPs) in cancers from human adults, but whether such subtype-specific RP mRNA signatures are also present in human pediatric cancers is currently unknown. In this study, we analyzed mRNA expression data from multiple large pediatric cancer datasets to test for heterogeneity in RP mRNA signatures. We found that different pediatric cancer types have different RP mRNA signatures, sometimes multiple RP mRNA signatures within the same pediatric cancer type, which can be subgroup/subtype-specific (e.g., in Medulloblastoma) or cell-of-origin-specific (e.g., in Acute Lymphoblastic Leukemia (ALL)). In B-cell ALL, we found two RP mRNA subtypes with significantly different prognoses. Consistent with our recent findings in adult cancers, the RP mRNA signature in pediatric cancer is heterogeneous and subtype-specific and may have clinical relevance.

## 1. Introduction

The ribosome is conventionally believed to have homogeneous composition [1] with fixed stoichiometry [2] among core ribosomal proteins (RPs). Consequently, unlike other genes, the RP mRNA pattern is conventionally expected to be relatively uniform apart from inter-sample variation in the total RP level. However, previous work by our group [3,4] and others [1,2,5,6,7] suggest the possibility that the ribosome may not be monolithic and may have a heterogeneous composition in response to extra/intracellular stimuli. Specifically, in a recent study [3], we observed subtype-specific mRNA and copy number variation (CNV) signatures of RPs in cancers from human adults. Among other results, we found that in human adults, different cancer types, and sometimes different subtypes of the same cancer type, often have different RP mRNA signatures [3], even after accounting for trivial inter-sample variation in total RP mRNA. Several adult cancer types were found to have multiple RP mRNA subtypes, with significant survival and genomic differences between these RP mRNA subtypes [3]. Whether such heterogeneity in RP mRNA signatures, with potential clinical relevance, is also present in human pediatric cancers is currently unknown.

Therefore, in this study, we decided to specifically focus on RP genes, and investigate patterns of RP signatures in pediatric cancers. We analyzed publicly available mRNA expression data from multiple large pediatric cancer datasets, namely, Therapeutically Applicable Research To Generate Effective Treatments (TARGET, N = 1322), Pediatric Brain Tumor Atlas from Children’s Brain Tumor Tissue Consortium (CBTTC, N = 996), and a large Medulloblastoma dataset (GSE85218, N = 763) from a recent study [8], to test for the presence of heterogeneous RP mRNA signatures beyond trivial inter-sample variation in total RP mRNA. We found that using the mRNA data of just 78 RP genes, properly normalized [3] to eliminate inter-sample variation in total RP mRNA, pediatric tumors can still be clearly clustered into cancer type/subtype-specific groups. Thus, contrary to conventional expectation, the RP mRNA pattern is not relatively uniform; instead, consistent with the possibility of heterogeneous ribosomal composition, the RP mRNA pattern is cancer type/subtype-specific. Just like adult cancers, different pediatric cancers, even those originating in the same tissue/organ, and sometimes different subgroups/subtypes of the same pediatric cancer, often have different RP mRNA signatures. Additionally, B-cell Acute Lymphoblastic Leukemia (ALL) has two distinct RP mRNA subtypes with significantly different prognoses. These results indicate the presence of heterogeneous RP mRNA signatures in pediatric cancers with potential clinical relevance, and suggests the possibility of different pediatric cancers having different ribosomal compositions.

## 2. Results

### 2.1. Subtype-Specific RP mRNA Signature in Pediatric Cancers from TARGET

**Figure 1:**
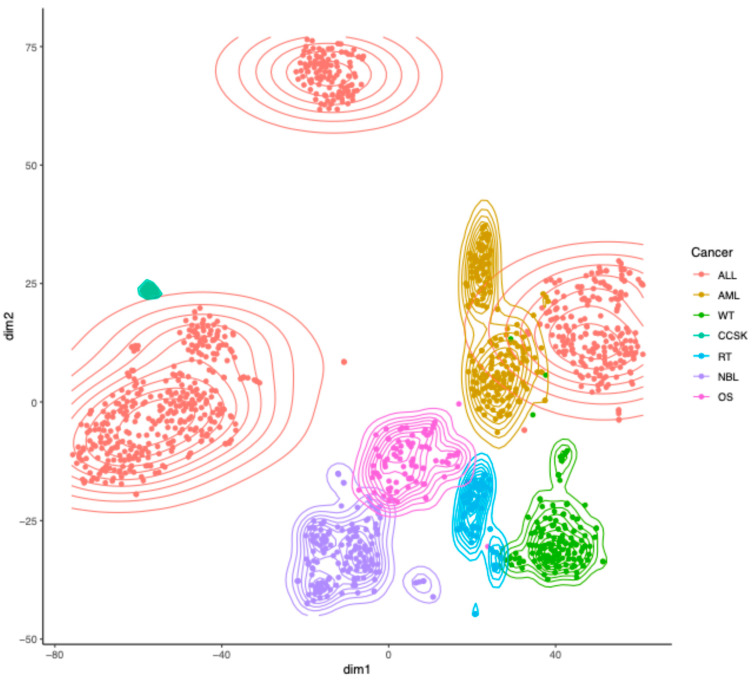
Heterogeneity in RP mRNA signatures in pediatric cancer from TARGET. Two-dimensional projection of properly normalized RP mRNA data from TARGET using t-SNE, with samples colored by cancer type. ALL = Acute Lymphoblastic Leukemia, AML = Acute Myeloid Leukemia, WT = Wilms Tumor, CCSK = Clear Cell Sarcoma of the Kidney, RT = Rhabdoid Tumor, NBL = Neuroblastoma, OS = Osteosarcoma.

The TARGET dataset (N = 1322) consisted of seven pediatric cancer types: two types of blood cancers, namely, Acute Lymphoblastic Leukemia (ALL, N = 679) and Acute Myeloid Leukemia (AML, N = 187); three types of kidney cancers, namely, Wilms Tumor (WT, N = 130), Clear Cell Sarcoma of the Kidney (CCSK, N = 13), and Rhabdoid Tumor (RT, N = 64); and two other cancer types, namely, Neuroblastoma (NBL, N = 161) and Osteosarcoma (OS, N = 88). ALL included T-cell ALL (T-ALL), B-cell ALL (B-ALL), and Acute Leukemia of Ambiguous Lineage (ALAL). Projecting the RP mRNA data, which were properly normalized (details in the Materials and Methods) to eliminate trivial inter-sample variation in total RP mRNA, to 2D using t-distributed Stochastic Neighbor Embedding (t-SNE) [9] showed multiple clusters in the TARGET dataset (Figure 1), indicating the presence of substantial heterogeneity in RP mRNA signatures in pediatric cancer. Coloring the samples by cancer type revealed that the clusters match cancer types (Figure 1), indicating that the observed heterogeneity in RP mRNA signatures is cancer type-specific. This finding was successfully validated using Self-Organizing Map (SOM) (Figure S1A).

**Figure 2:**
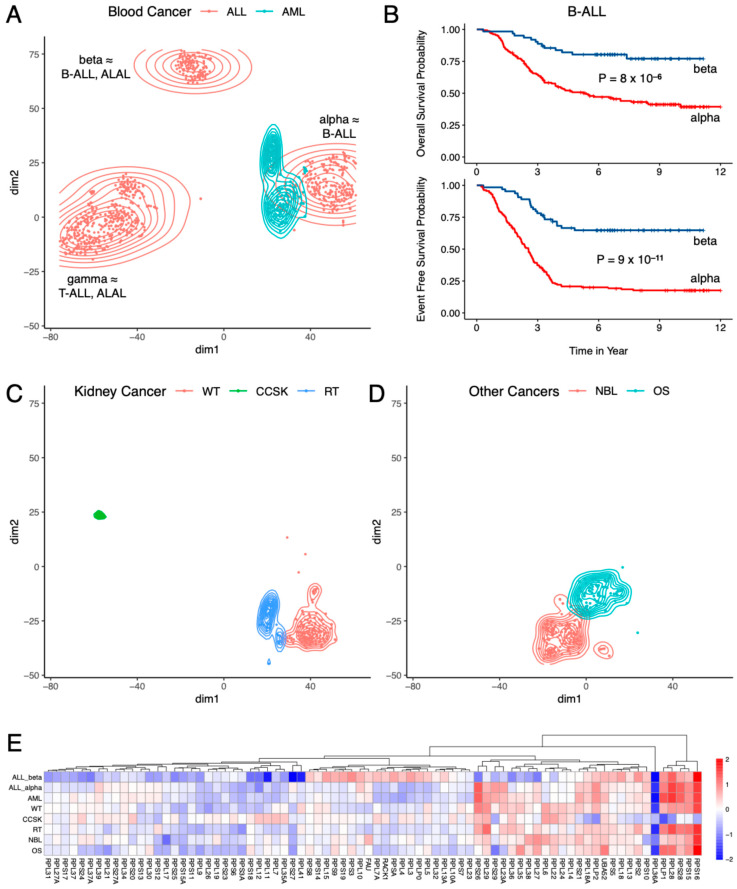
Subtype-specific RP mRNA signature in pediatric cancers from TARGET. (**A**) Two types of blood cancers had different RP mRNA signatures, and ALL had three distinct RP mRNA subtypes. (**B**) Two RP mRNA subtypes of B-cell ALL had significantly different prognoses. (**C**) Three types of kidney cancers had different RP mRNA signatures. (**D**) Two other cancer types from TARGET had different RP mRNA signatures. (**E**) log_2_ fold change in each cancer type compared to the cluster with the highest number of samples (cluster gamma of ALL). ALL = Acute Lymphoblastic Leukemia, B-ALL = B-cell Acute Lymphoblastic Leukemia, T-ALL = T-cell Acute Lymphoblastic Leukemia, ALAL = Acute Leukemia of Ambiguous Lineage, AML = Acute Myeloid Leukemia, WT = Wilms Tumor, CCSK = Clear Cell Sarcoma of the Kidney, RT = Rhabdoid Tumor, NBL = Neuroblastoma, OS = Osteosarcoma.

Closer inspection of the t-SNE results (Figure 2) showed that different pediatric cancer types originating in the same tissue/organ had different RP mRNA signatures. In blood cancer (Figure 2A) for example, ALL and AML clustered separately, and were clearly distinguishable from each other. But more interestingly, there were three distinct clusters of ALL, cleanly separated from each other (Figure 2A), indicating the presence of three RP mRNA subtypes in ALL, which we named alpha, beta, and gamma. This finding was successfully validated using SOM (Figure S1B). Figure S2 shows the top RPs differentially expressed between the three clusters of ALL. An analysis of the cell of origin data showed that cluster gamma (N = 361) consisted of T-ALL (265/345 = 76.8%) and ALAL (74/345 = 21.4%), cluster beta (N = 111) consisted of B-ALL (65/108 = 60.2%) and ALAL (43/108 = 39.8%), and cluster alpha (N = 207) was mostly B-ALL (206/207 = 99.5%). This indicates that T-ALL has a different RP mRNA signature to that of B-ALL, but, more importantly, that both B-ALL and ALAL have two distinct RP mRNA subtypes each. Figures S3 and S4 show the top RPs differentially expressed between the two clusters of ALAL and the two clusters of B-ALL, respectively. Although cluster alpha of B-ALL had significantly more recurrent tumor samples than cluster beta of B-ALL (65/206 = 31.6% vs. 4/65 = 6.2%, *p* = 1.4 × 10^−5^), most of the B-ALL samples in both clusters were primary tumor samples.

While there was no statistically significant difference in overall survival, event-free survival, age, sex, initial white blood cell (WBC) count, and World Health Organization (WHO) classification between the two clusters of ALAL, the two clusters of B-ALL had significantly different prognoses (Figure 2B): cluster alpha of B-ALL (N = 158) had a significantly shorter overall survival (median 5.39 years vs. not reached, *p* = 8 × 10^−6^) and event-free survival (median 2.54 years vs. not reached, *p* = 9 × 10^−11^) than those of cluster beta of B-ALL (N = 64). Even though there was no statistically significant difference between the two clusters of B-ALL for race, ethnicity, sex, presence of Down syndrome, WBC count, and central nervous system (CNS) involvement at diagnosis, the difference in prognosis manifested quite early, as 53.5% (83/155) of B-ALL cases from cluster alpha had minimum residual disease on day 29 of induction therapy compared to 27.9% (17/61) of B-ALL cases from cluster beta (*p* = 8 × 10^−4^). Subsequently 75.3% (119/158) of B-ALL cases from cluster alpha had bone marrow relapse compared to 51.6% (16/31) of B-ALL cases from cluster beta (*p* = 0.015), even though there was no statistically significant difference in CNS, testes, or other site relapse.

High hyperdiploidy (DNA index ≥ 1.16) [10], trisomy of chromosomes 4 and 10 [11], and *ETV6-RUNX1* fusion [12] are known to be associated with better prognosis in B-ALL. Surprisingly, cluster alpha of B-ALL had worse prognosis than cluster beta despite being significantly more enriched in high hyperdiploidy (29/158 = 18.4% vs. 4/65 = 6.2%, *p* = 0.022) and trisomy of chromosomes 4 and 10 (17/138 = 12.3% vs. 2/64 = 3.1%, *p* = 0.040), and considerably more enriched in *ETV6-RUNX1* fusion (12/130 = 9.2% vs. 1/62 = 1.6%, *p* = 0.064). No statistically significant difference was observed between the two clusters for *BCR-ABL1* fusion, *TCF3-PBX1* fusion, and *KMT2A* rearrangement. Age at diagnosis was significantly higher in cluster alpha (N = 158) of B-ALL than cluster beta (N = 65) of B-ALL (median 6.48 years vs. 4.52 years, *p* = 0.043), which may partially explain the worse prognosis of cluster alpha as older age at diagnosis is associated with poor prognosis in B-ALL [13]. The difference in prognosis between the two RP mRNA subtypes of B-ALL suggests that heterogeneity in RP mRNA signature may have clinical relevance. The RP mRNA subtype was statistically significant (*p* = 1.5 × 10^−4^, 2.8 × 10^−8^, respectively) in the multivariate Cox proportional hazard analysis of both overall survival (Figure S5A) and event-free survival (Figure S5B), suggesting that RP mRNA subtype is an independent prognostic factor in B-ALL. Consistently, even among B-ALL cases that had traditionally good prognostic markers (i.e., high hyperdiploidy, trisomy of chromosomes 4 and 10, and *ETV6-RUNX1* fusion), cluster alpha (N = 43) had significantly shorter (*p* = 0.035) event-free survival (Figure S5C) than cluster beta (N = 5). However, within cluster alpha of B-ALL, cases with traditionally good prognostic markers (N = 43) still had a significantly longer (*p* = 0.022) overall survival (Figure S5D) than the remaining cases (N = 115).

Like blood cancer (Figure 2A), different types of pediatric kidney cancers (Figure 2C) also had different RP mRNA signatures, as evident from the fact that WT, CCSK, and RT all clustered separately and were clearly distinguishable from each other. Figure S6 shows the top RPs differentially expressed between the three types of kidney cancers. Other pediatric cancer types in TARGET, namely, NBL and OS, clustered separately from each other (Figure 2D), and separately from blood and kidney cancers (Figure 1), indicating that they too have their own distinct RP mRNA signature. Finally, we compared each cancer type against the cluster with the highest number of samples, namely, cluster gamma of ALL, and Figure 2E shows the resulting fold changes in log_2_ scale. It is visually apparent from Figure 2E that different pediatric cancer types in TARGET have different RP mRNA signatures, although the magnitude of the differences are often small (<2-fold) for most RPs.

### 2.2. Subtype-Specific RP mRNA Signature in Pediatric Brain Cancers

**Figure 3:**
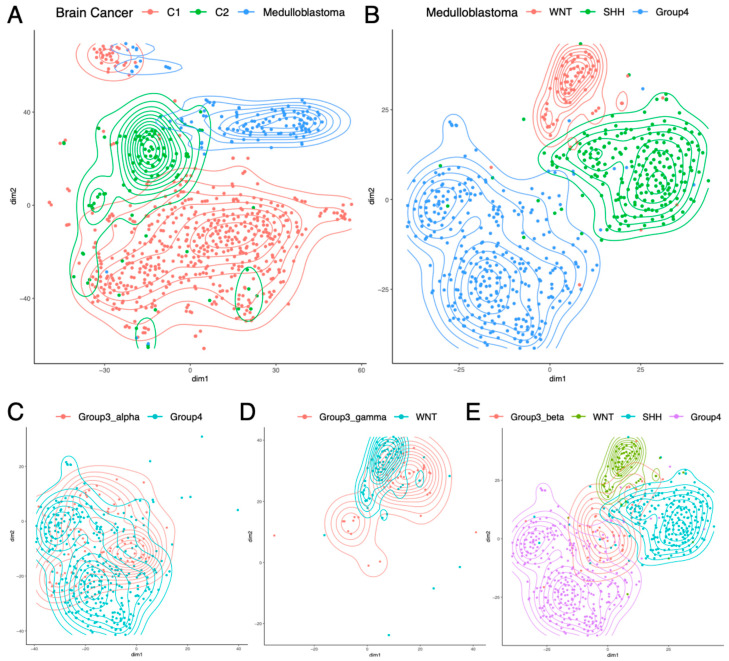
Subtype-specific RP mRNA signature in pediatric brain cancers. (**A**) Different types of brain cancers had different RP mRNA signatures. C1: Low-grade and high-grade Glioma/Astrocytoma, Ependymoma, Ganglioglioma, and Dysembryoplastic Neuroepithelial tumor; C2: Craniopharyngioma, Atypical Teratoid Rhabdoid Tumor, and Meningioma. (**B**) Of the four subgroups of Medulloblastoma, three had distinct RP mRNA signatures. (**C**) Group3_alpha subtype had an RP mRNA signature similar to Group4 subgroup. (**D**) Group3_gamma subtype had an RP mRNA signature similar to WNT subgroup. (**E**) Group3_beta subtype had a distinct RP mRNA signature different from the other three subgroups.

The TARGET dataset did not include pediatric brain cancers, so, next, we analyzed pediatric brain cancers from CBTTC (N = 996). Although this dataset included 38 types of pediatric brain cancers, most cancer types had very few samples, and only nine cancer types had 25 or more samples. The properly normalized RP mRNA data were projected to 2D using t-SNE [9], and Figure 3A shows the samples from these nine cancer types. While Medulloblastoma (N = 123) formed a separate cluster of its own, the other eight cancer types organized themselves into two largely distinct groups. This finding was successfully validated using SOM (Figure S1C). One group (C1) consisted of low-grade (N = 254) and high-grade (N = 131) Glioma/Astrocytoma, Ependymoma (N = 93), Ganglioglioma (N = 49), and Dysembryoplastic Neuroepithelial tumor (N = 25), which overlapped with each other. The other group (C2) consisted of Craniopharyngioma (N = 36), Atypical Teratoid Rhabdoid Tumor (N = 30), and Meningioma (N = 29), which overlapped with each other. Figure S7 shows the top RPs differentially expressed between C1, C2, and Medulloblastoma. Within C1, there was some separation between low-grade (I/II) and high-grade (III/IV) Glioma/Astrocytoma, but there was too much overlap, which justified their inclusion within the same group. These results indicate that like pediatric blood cancer (Figure 2A) and pediatric kidney cancer (Figure 2C), pediatric brain cancer (Figure 3A) also shows cancer type-specific RP mRNA signatures.

Subsequently, we analyzed a large Medulloblastoma dataset (GSE85218, N = 763) from a recent study [8]. The dataset consisted of four subgroups of Medulloblastoma (WNT, SHH, Group3, and Group4), where each subgroup had two to four subtypes (alpha, beta, gamma, delta). Two-dimensional projection of the properly normalized RP mRNA data using t-SNE [9] showed that three of the four subgroups (WNT, SHH, Group4) had distinct RP mRNA signatures (Figure 3B). This finding was successfully validated using SOM (Figure S1D). Figure S8 shows the top RPs differentially expressed between these three subgroups. These three subgroups did not show subtype-specific RP mRNA signatures, i.e., the two subtypes of WNT had similar RP mRNA signatures, the four subtypes of SHH had similar RP mRNA signatures, and the three subtypes of Group4 had similar RP mRNA signatures. However, the subgroup Group3 showed subtype-specific RP mRNA signatures, where Group3_alpha subtype had an RP mRNA signature similar to that of Group4 subgroup (Figure 3C), and the Group3_gamma subtype had an RP mRNA signature similar to that of the WNT subgroup (Figure 3D), while the Group3_beta subtype had its own RP mRNA signature distinct from that of the other three subgroups (Figure 3E). Figure S9 shows the top RPs differentially expressed in Group3_beta compared to the other three subgroups. These results indicate the presence of four distinct RP mRNA signatures in Medulloblastoma that are subgroup/subtype-specific: one shared by WNT and Group3_gamma, one unique to SHH, another shared by Group4 and Group3_alpha, and another unique to Group3_beta.

## 3. Discussion

Emerging observations of an association between mutations in ribosomal protein coding genes and pediatric cancers have begun to demonstrate the importance of ribosomal biology in pediatric tumor formation [14,15]. For example, 10% of pediatric T-ALLs have mutations in ribosomal proteins [16]. Along the same lines, select ribosomal proteins have been found to be differentially expressed in different pediatric cancers like Medulloblastoma [17,18]. However, whether pediatric cancer development is a result of functional disruption of selective ribosomal proteins or ribosomal biology that has been collectively hijacked or disrupted, impacting cancer progression, remains unclear. In a recent study [3], we observed subtype-specific RP mRNA signatures in cancers from human adults, but whether such subtype-specific RP mRNA signatures are also present in human pediatric cancers is currently unknown.

In the present study, by analyzing mRNA expression data from several large pediatric cancer datasets, we showed that subtype-specific RP mRNA signatures are also present in pediatric cancers. Reliance on RNA-seq data alone is a shortcoming of this study. However, in our previous study [3], we showed that ribosome profiling data for RPs is highly correlated with RNA-seq data for RPs in human and rodent tissues and cell cultures, so we believe the use of RNA-seq data may be informative for the purpose of this study.

Broadly, the results of the present study show that different pediatric cancer types have different RP mRNA signatures. Specifically, in the case of blood cancer, kidney cancer, and brain cancer, the results show that different pediatric cancer types originating in the same tissue/organ usually have different RP mRNA signatures, although sometimes multiple cancer types can organize into a single RP mRNA group (e.g., C1 or C2 in brain cancer). Differences in RP mRNA signatures between cancer types may reflect the RP mRNA signatures of the normal cell of origin, or changes associated with oncogenic transformation, or the current epigenetic state of the cancer. Further studies are required to better understand the biological underpinnings of these signatures.

Some pediatric cancer types have multiple RP mRNA signatures that can be subgroup-specific as in Medulloblastoma, or cell-of-origin-specific as in ALL. B-ALL is particularly interesting, as it has two distinct RP mRNA signatures despite having the same cell of origin, which indicates the presence of two RP mRNA subtypes in B-ALL. We observed a significant difference in prognosis between the two RP mRNA subtypes of B-ALL, which suggests that heterogeneity in RP mRNA signatures may have clinical relevance. In fact, the RP mRNA subtype was an independent prognostic factor in B-ALL, independent of the age at diagnosis and presence of traditionally good prognostic markers (*ETV6-RUNX1* fusion [12], high hyperdiploidy [10], and trisomy of chromosomes 4 and 10 [11]), as well as presence of minimum residual disease on day 29 of induction therapy.

There is however an important caveat: TARGET specifically selected B-ALL cases that were either high-risk or had an early bone marrow relapse for RNA-seq, so the B-ALL cohort analyzed here is a pre-selected group that only includes high-risk cases and early relapse cases and thus may not be representative of B-ALL in general. Within this already pre-selected cohort of poor-prognosis B-ALL cases, we found two RP mRNA subtypes –cluster alpha and cluster beta (Figure 2A)—that had significantly different prognoses. Cluster alpha had a worse prognosis despite being enriched in the traditionally good prognostic markers listed above. However, this does not mean that traditionally good prognostic markers are associated with poor prognosis, as presumably most cases with good prognostic markers were low-risk and had late relapse and consequently were not selected by TARGET for RNA-seq and thus could not be analyzed here.

Among the sequenced B-ALL cases that had these traditionally good prognostic markers, cluster alpha had significantly shorter event-free survival than cluster beta, suggesting that cluster alpha identifies a subset of B-ALL that, despite harboring favorable genetic lesions, behaves aggressively. Cluster alpha however does not completely negate the effect of these traditionally good prognostic markers, as even within cluster alpha of B-ALL, cases with good prognostic markers had significantly longer overall survival than the remaining cases. However, given the selection bias, the analysis should be repeated in an unselected set of B-ALL in future studies to confirm these results.

The biological mechanism underlying the observed survival difference between the two RP mRNA subtypes of B-ALL remains unknown. It is possible that the two clusters have two different ribosomal composition, and that ribosomes with different compositions are more efficient at making different sets of proteins. If this is true, cluster alpha of B-ALL may be more efficient at making one set of proteins while cluster beta of B-ALL may be more efficient at making another set of proteins, leading to the two clusters having different fitness levels under chemotherapy. Alternatively, there may be some other mechanism that is responsible for the significant difference in prognosis between the two clusters of B-ALL.

These observations in pediatric cancers are consistent with what we observed in various adult cancers in a recent study [3]. Like pediatric B-ALL in the present study, six adult cancer types in our previous study [3]—low-grade glioma, skin cutaneous melanoma, uveal melanoma, bladder urothelial carcinoma, colorectal adenocarcinoma, and prostate adenocarcinoma—had RP mRNA subtypes with significantly different disease-specific survival or disease-free interval. Thus, to summarize, while the magnitude of the differences between RP mRNA signatures of different pediatric cancer types is often small (<2-fold) for most RPs, which is also the case in adult cancers [3], subtype-specific RP mRNA signatures are also present in pediatric cancers.

This is a novel and potentially important finding in pediatric cancer and in the potential role of ribosomal composition in different cell states. These results raise the possibility that ribosomal composition may be tuned for specific transcriptional programs, and that these may be altered in specific cancers. Experimental validation of these findings as well as functional studies to assess the effect of heterogeneous RP mRNA signatures on tumor properties are warranted. Subsequent studies are required to (i) validate the prognostic effect of RP mRNA signatures in larger prospective cancer datasets in the future; (ii) validate RP mRNA patterns seen in pediatric cancers with analyses of protein expression of RP genes, which will require obtaining fresh samples for protein analysis; (iii) answer the question of whether different patterns of RP mRNA seen in cancer types/subtypes drive key aspects of the malignant phenotype by genetically manipulating RP mRNA levels in primary cancer organoid and determining effect on tumor growth, survival and metastases; (iv) determine whether the RP mRNA clusters seen represent different cells of origin. For pediatric cancers whose incidence is enriched in early life, this may reflect early developmental cells of origin that are less present in adults and may suggest that RP gene use may be important in early development. This can be tested through an analysis of RP mRNA patterns during early development using mouse models.

The results of the present study suggest the possibility that different pediatric cancer types may have different ribosomal compositions and adds to a growing body of work [1,2,3,4,5,6,7] that suggests the possibility of heterogeneous ribosomal composition. It should be noted that differences in RP mRNA signature do not automatically equate to differences in ribosomal composition, as translational regulation or post-transcriptional modification can modulate actual ribosomal composition. While functional heterogeneity of ribosomes is not directly proven here, the heterogeneity of RP mRNA signatures is consistent with the possibility of different ribosomal composition being present in different pediatric cancers.

## 4. Materials and Methods

### 4.1. Datasets and Normalization

RNA-seq read count (HTSeq) data and clinical data of 1322 tumor samples from TARGET were obtained from NCI GDC (https://portal.gdc.cancer.gov) (accessed on 4 May 2021 and 25 October 2025, respectively). The read count data was restricted to 78 non-sex-specific RPs known to be functional in humans [19], and then divided by gene length. Since different samples may have different levels of total RP mRNA, this reads per kilobase data was then normalized so that the sum over all 78 RP genes was the same for each sample. As previously explained [3], this normalization is necessary to eliminate trivial inter-sample variation in total RP mRNA, so that we can explore true variations in ratios of RP mRNA levels. RNA-seq expression (FPKM) data of 996 brain tumor samples from CBTTC were obtained from UCSC Xena (https://xenabrowser.net/datapages/?cohort=Pediatric%20Brain%20Tumor%20Atlas%3A%20CBTTC) (accessed on 13 June 2020), then restricted to the 78 non-sex-specific RPs, and then normalized so that the sum over all 78 RP genes was the same for each sample. Gene expression (microarray) data of 763 Medulloblastoma samples from a recent study [8] was obtained from Gene Expression Omnibus (https://www.ncbi.nlm.nih.gov/geo/query/acc.cgi?acc=GSE85218) (accessed on 15 June 2020), and then linearized as x → 2^x^. This linearized data was restricted to 67 RP genes (as expression data was unavailable for the remaining 11 RP genes), and then normalized so that the sum over all RP genes was the same for each sample.

### 4.2. Analysis of Normalized Data

Each of these normalized datasets were (separately) analyzed by t-distributed Stochastic Neighbor Embedding (t-SNE) [9] in matlab via the function ‘tsne’ using the ‘exact’ algorithm with default parameters, and the distance metric $d\left(i,j\right)=\Sigma_{r}\left|{log}_{2}\left(FC\left(i,j,r\right)\right)\right|\;$, where FC(i,j,r) is the fold change for the rth RP for sample pair (i,j), and the index r takes $N_{r}$ values corresponding to the number of RPs and the indices i,j range from 1 to $N_{s}$ (the number of samples), as previously described [3]. In the 2D projection from t-SNE, samples were colored by cancer type/subgroup/subtype to identify cancer type/subgroup/subtype-specific clusters. The clusters identified by t-SNE were validated using a different clustering method: Self-Organizing Map (SOM). As previously described [3], samples were mapped to hexagonal nodes using SOM in a 2D grid using the ‘kohonen’ package in R (‘rlen’ was set to 10,000 and other parameters had default values), and the mapped nodes in SOM were colored by t-SNE clusters using a majority rule: if >50% of the samples mapping to an SOM node originated from a t-SNE cluster, that SOM node was assigned the color of that t-SNE cluster. Thus, a co-localization of nodes of the same color in SOM would validate the clusters identified by t-SNE. The package ‘pheatmap’ in R was used for hierarchical clustering, and the packages ‘survival’ and ‘survminer’ in R were used for survival analysis, including the multivariate Cox proportional hazard analysis. The two-sided Wilcoxon rank sum test was used for comparison of continuous variables (including gene expression), while the two-sided Fisher’s exact test was used for comparison of categorical variables. Statistical significance was assessed at *p* < 0.05.

## 5. Conclusions

This study shows that different pediatric cancer types have different RP mRNA signatures, and some pediatric cancer types like Acute Lymphoblastic Leukemia (ALL) and Medulloblastoma have multiple RP mRNA subtypes. There was a significant difference in prognoses between the RP mRNA subtypes of B-cell ALL, and the RP mRNA subtype was an independent prognostic factor in B-cell ALL. These results indicate the presence of heterogeneous RP mRNA signatures in pediatric cancers with potential clinical relevance and is consistent with our previous findings in adult cancers.

## Data Availability

Links to publicly available datasets analyzed in this study are provided in the Section 4.1.

## Supplementary Materials

The following supporting information can be downloaded at https://www.mdpi.com/article/10.3390/ijms262412036/s1.
