# Supplementary material for: Subtype-Specific mRNA Signatures of Human Ribosomal Proteins in Pediatric Cancers
Source: Int J Mol Sci. 2025 Dec 14;26(24):12036. doi: 10.3390/ijms262412036 (PMC12733358; doi:10.3390/ijms262412036)
Supplement: Supplementary file 1 [file ijms-26-12036-s001.zip › ijms-3995694-supplementary.pdf]

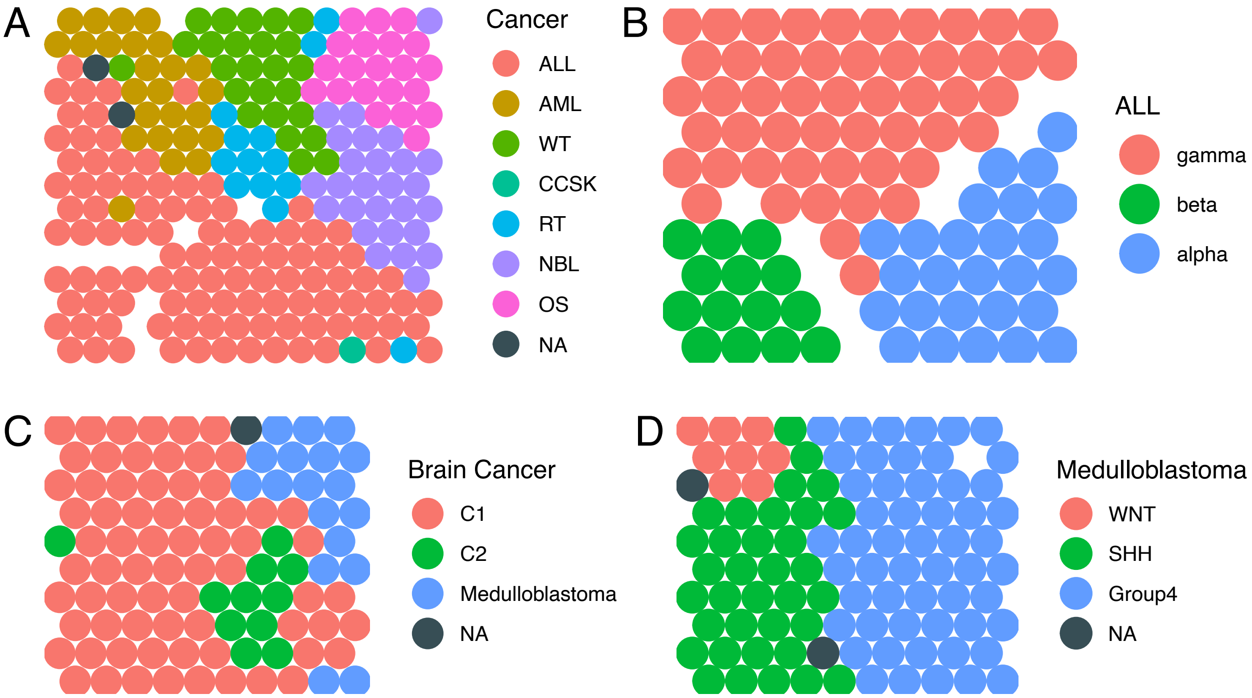

Figure S1. Validation of the Ribosomal Protein (RP) mRNA clusters identified by t-distributed Stochastic Neighbor Embedding (t-SNE) using Self Organizing Map (SOM). Validation of (A) cancer type specific RP mRNA clusters from Figure 1, (B) three RP mRNA clusters of Acute Lymphoblastic Leukemia (ALL) from Figure 2A, (C) RP mRNA clusters of brain cancer from Figure 3A, (D) RP mRNA clusters of Medulloblastoma from Figure 3B. ALL = Acute Lymphoblastic Leukemia, AML = Acute Myeloid Leukemia, WT = Wilms Tumor, CCSK = Clear Cell Sarcoma of the Kidney, RT = Rhabdoid Tumor, NBL = Neuroblastoma, OS = Osteosarcoma. C1 = low-grade and high-grade Glioma/Astrocytoma, Ependymoma, Ganglioglioma, and Dysembryoplastic Neuroepithelial tumor; C2: Craniopharyngioma, Atypical Teratoid Rhabdoid Tumor, and Meningioma. Nodes marked NA had no majority and empty nodes had no samples mapped. Although drawn as circles, the nodes are actually hexagonal.

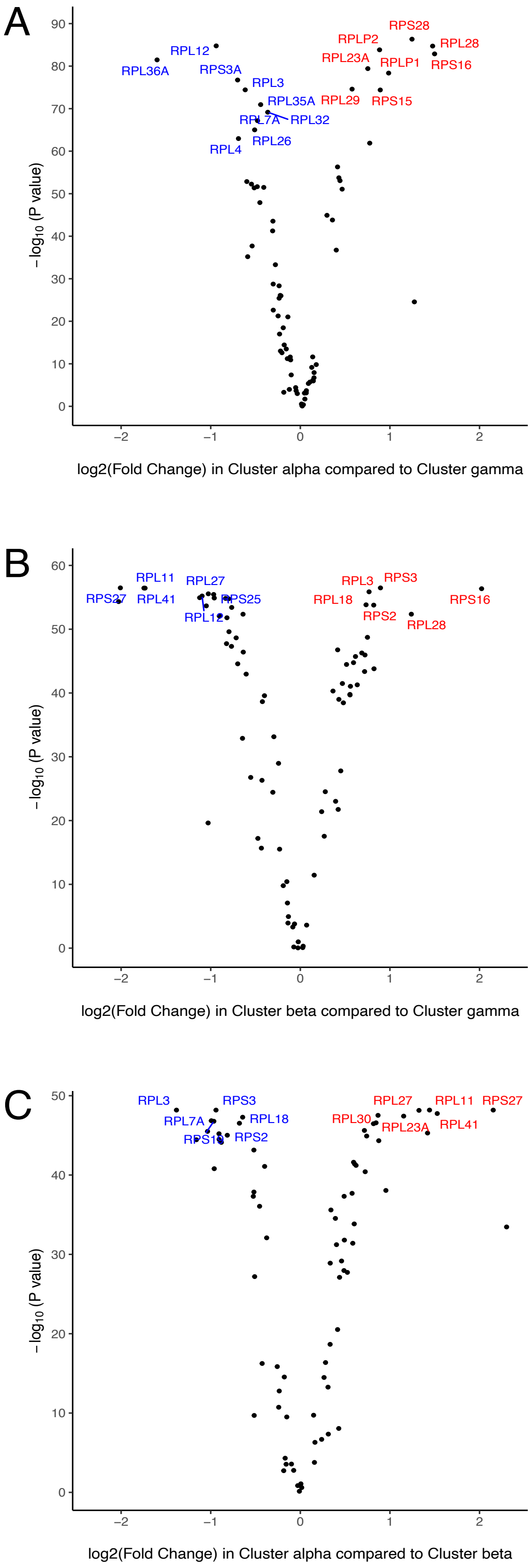

Figure S2. RPs differentially expressed between the three clusters of Acute Lymphoblastic Leukemia (ALL).

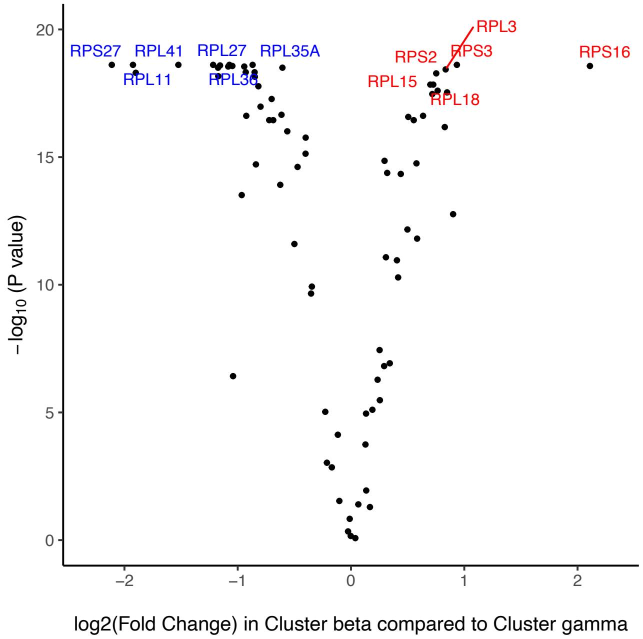

Figure S3. RPs differentially expressed between the two clusters of Acute Leukemia of Ambiguous Lineage (ALAL).

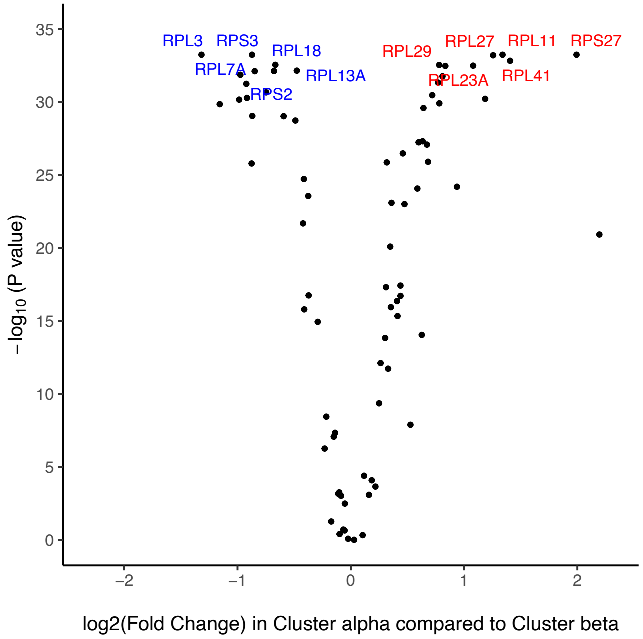

Figure S4. RPs differentially expressed between the two clusters of B-cell Acute Lymphoblastic Leukemia (B-ALL).

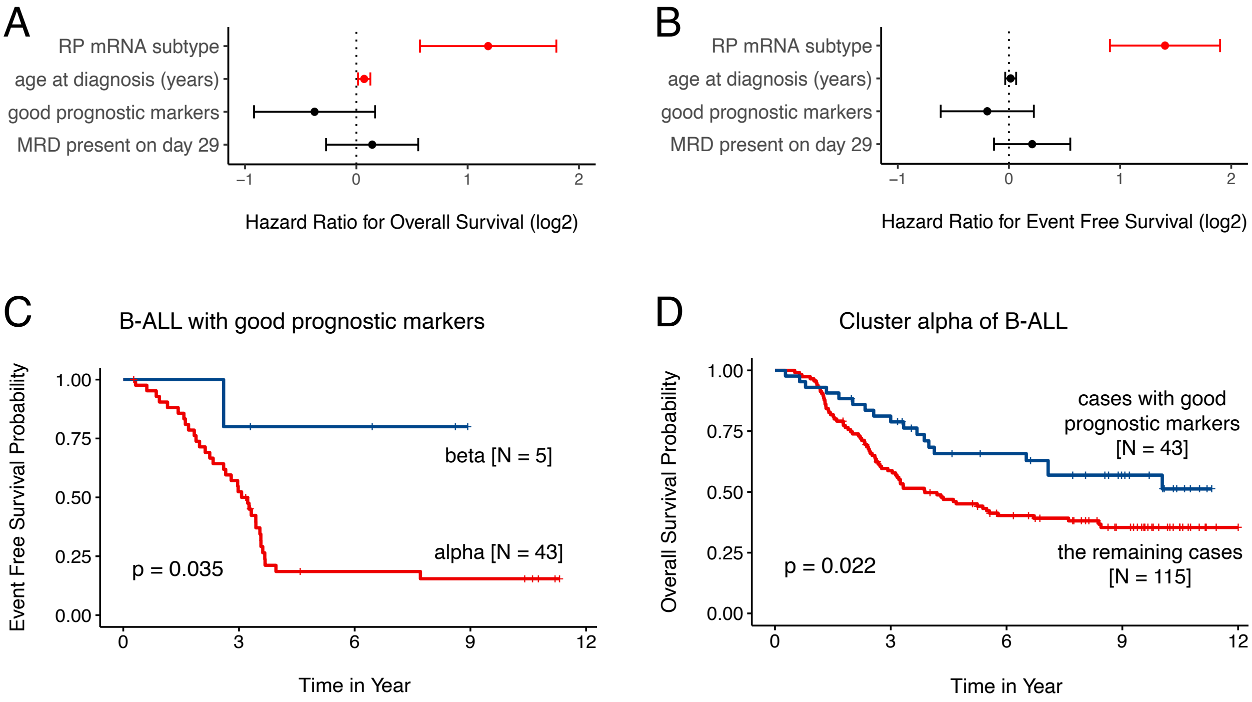

Figure S5. Detailed survival analysis of B-cell Acute Lymphoblastic Leukemia (B-ALL). Multivariate Cox proportional hazard analysis of (A) overall survival and (B) event free survival. (C) B-ALL cases with traditionally good prognostic markers stratified by RP mRNA subtype. (D) Cluster alpha of B-ALL stratified by the presence of traditionally good prognostic markers. Good prognostic markers = *ETV6-RUNX1* fusion, high hyperdiploidy (DNA index ≥ 1.16), and trisomy of chromosomes 4 and 10; MRD = minimum residual disease.

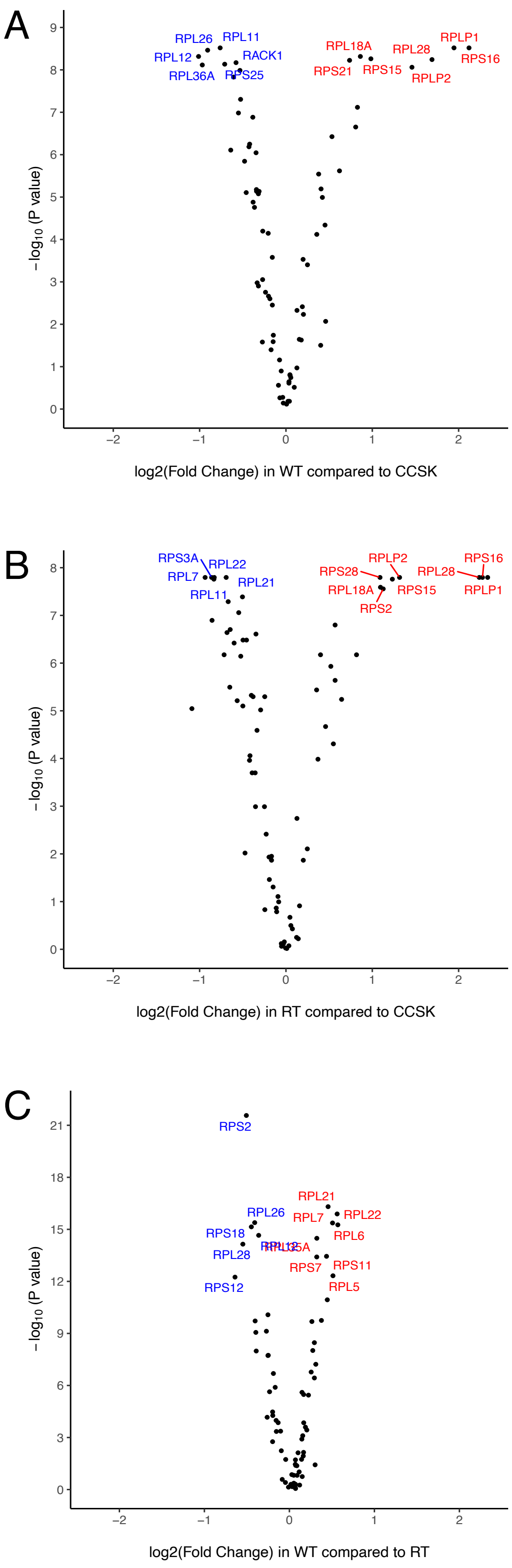

Figure S6. RPs differentially expressed between three types of kidney cancers. WT = Wilms Tumor, CCSK = Clear Cell Sarcoma of the Kidney, RT = Rhabdoid Tumor.

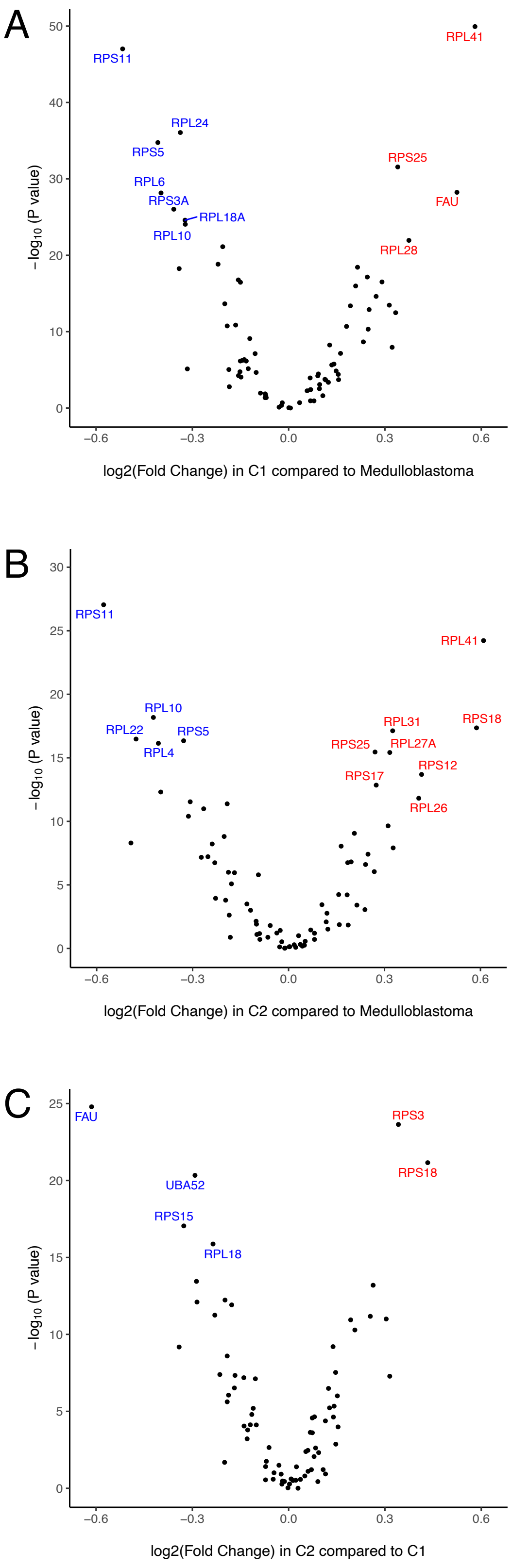

Figure S7. RPs differentially expressed between different types of brain cancers. C1: low-grade and high-grade Glioma / Astrocytoma, Ependymoma, Ganglioglioma, and Dysembryoplastic Neuroepithelial tumor; C2: Craniopharyngioma, Atypical Teratoid Rhabdoid Tumor, and Meningioma.

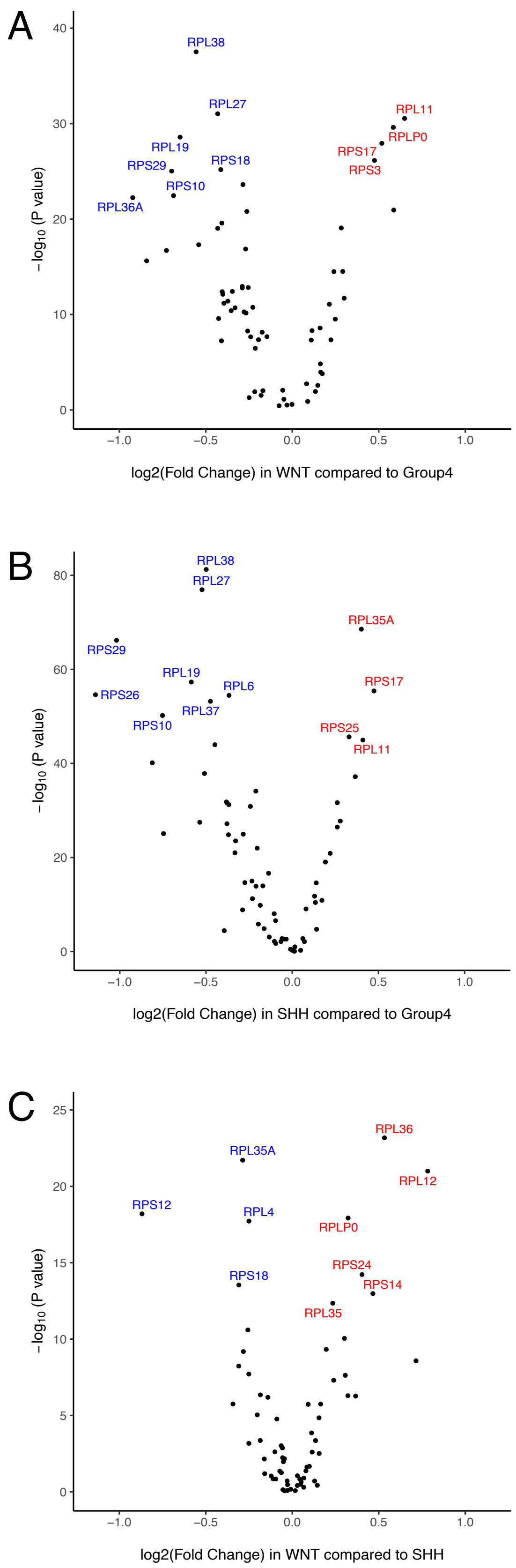

Figure S8. RPs differentially expressed between three subgroups of Medulloblastoma.

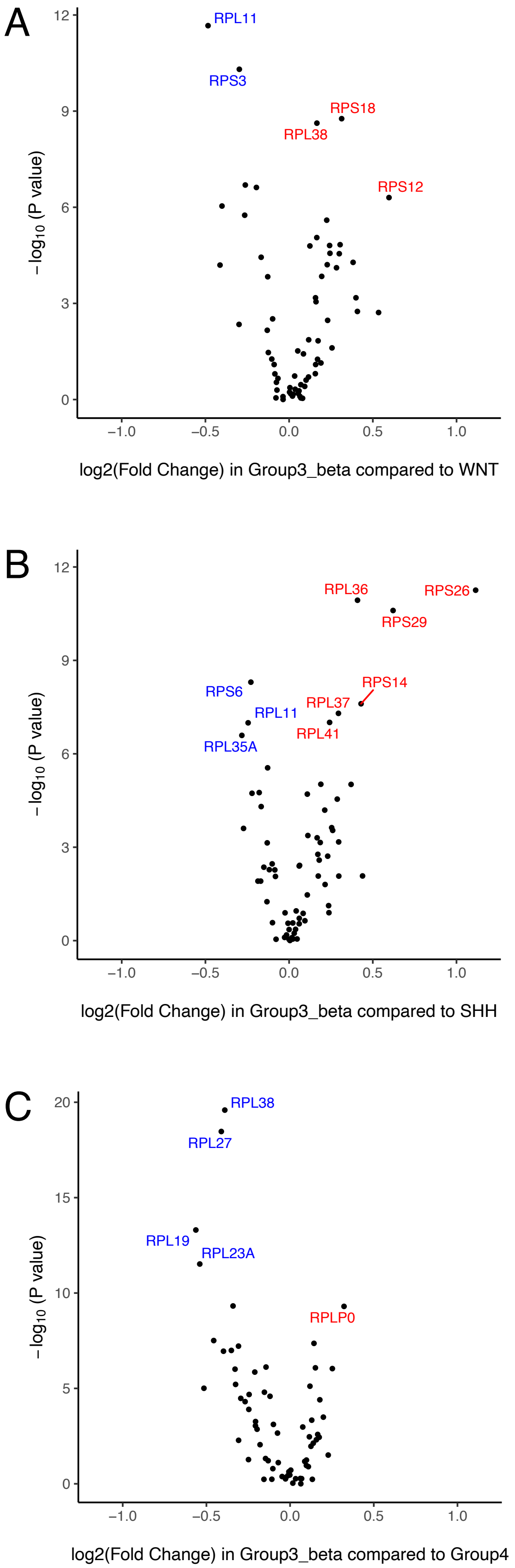

Figure S9. RPs differentially expressed in Group3_beta subtype compared to three subgroups of Medulloblastoma.
